# Self‐Assembled nanoparticles of natural bioactive molecules enhance the delivery and efficacy of paclitaxel in glioblastoma

**DOI:** 10.1111/cns.14528

**Published:** 2023-12-04

**Authors:** Yong Li, Qingyu Zhao, Xinyi Zhu, Long Zhou, Ping Song, Baohui Liu, Daofeng Tian, Qianxue Chen, Jiangbing Zhou, Gang Deng

**Affiliations:** ^1^ Department of Neurosurgery Renmin Hospital of Wuhan University Wuhan Hubei P.R. China; ^2^ Department of Neurosurgery Yale University New Haven Connecticut USA

**Keywords:** chemoresistance, glioblastoma, natural bioactive molecules, paclitaxel, P‐glycoprotein, self‐assembled nanoparticles

## Abstract

**Background:**

Glioblastoma (GBM) is the most common primary malignant tumor in the central nervous system. Paclitaxel (PTX) is a well‐established and highly effective anti‐cancer drug for peripheral solid tumors. However, the application of PTX in GBM is hindered by several limitations, including poor water solubility, restricted entry across the blood–brain barrier (BBB), and enhanced excretion by efflux transporters. P‐glycoprotein (P‐gp) is a crucial efflux transporter that is abundantly present in cerebral vascular endothelial cells and GBM cells. It plays a significant role in the exocytosis of PTX within tumor tissues.

**Methods:**

Recently, we have developed a novel technique for creating self‐assembled nanoparticles utilizing a range of natural bioactive molecules. These nanoparticles can encapsulate insoluble drugs and effectively cross the BBB. In additional, we revealed that certain nanoparticles have the potential to act as P‐gp inhibitors, thereby reducing the excretion of PTX. In this study, we conducted a screening of bioactive molecular nanoparticles to identify those that effectively inhibit the function of P‐gp transporters.

**Results:**

Among the candidates, we identified ursolic acid nanoparticles (UA NPs) as the P‐gp inhibitors. Furthermore, we prepared co‐assembled UA NPs embedded with paclitaxel, referred to as UA‐PTX NPs. Our results demonstrate that UA‐PTX NPs can enhance the blood concentration of PTX, facilitate its entry into the BBB, and inhibit the function of P‐gp, resulting in a decrease in the excretion of PTX. This discovery effectively addressed the above three issues associated with the use of PTX in glioma treatment.

**Conclusions:**

UA‐PTX NPs demonstrate strong anti‐tumor effects and show great potential for treating GBM.

## Graphical abstract

Enhancing the anti‐tumor effect of PTX in GBM through self‐assembled nanoparticles of natural bioactive molecules with anti P‐gp activity, leading to improved water‐solubility, BBB permeability, and chemosensitivity of PTX.

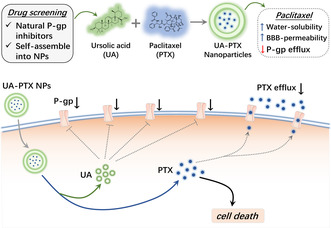

## 1 INTRODUCTION

Glioblastoma (GBM) is the most common primary malignant tumor in the central nervous system.^1^ Temozolomide (TMZ) is currently the only first‐line drug for treating GBM, and when combined with radiotherapy, it can partially extend the survival of GBM patients. However, even with the standard treatment regimen of microsurgical resection, postoperative radiotherapy, and concurrent adjuvant TMZ chemotherapy, the 5‐year survival rate for GBM patients remains below 10%.^2^ One of the major reasons for treatment failure is believed to be the development of chemoresistance to TMZ.^3^ Therefore, there is an urgent need to identify alternative anticancer drugs for GBM patients who are resistant to TMZ.

Paclitaxel (PTX) is a powerful natural anti‐cancer drug derived from red shank plants. It is widely used in clinical treatment for breast cancer, ovarian cancer, and other tumors.^4^ PTX works by interacting with tubulin in cells. It binds directly to tubulin and stabilize the microtubule structure, which prevents cell division. This ultimately leads to cell death as the cells remain stuck in the G2 or M phase.^5^ In laboratory experiments, PTX has shown to be approximately 1400 times more effective than TMZ in inhibiting GBM cells.^6^ However, its application in treating GBM is limited due to several factors: (1) PTX has poor water solubility; (2) it is unable to cross the blood–brain barrier (BBB); (3) efflux transporters actively remove PTX from the tumor, reducing its effectiveness.^7^, ^8^

Multidrug resistance is a significant issue in the field of cancer treatment, often leading to the failure of chemotherapy.^9^ The molecular mechanism behind tumor multidrug resistance is highly complex, with the overexpression of ATP binding cassette (ABC) transporters playing a crucial role. These ABC transporters, also known as efflux transporters, primarily include P‐glycoprotein (P‐gp/ABCB1), MRP1, and BCRP.^8^ Among these transporters, P‐gp was the first to be discovered and is found to be highly expressed in cerebral vascular endothelial cells and brain tumors. Notably, PTX is mainly eliminated through P‐gp.^10^

Numerous therapeutic effects in various diseases have been attributed to bioactive molecules derived from natural sources.^11^, ^12^, ^13^, ^14^ Continual reports have identified a significant number of natural bioactive molecules that exhibit inhibitory properties against P‐gp.^15^, ^16^, ^17^ These bioactive molecules are widely distributed in nature, offering a vast pool of resources for the efficient and non‐toxic screening and exploration of P‐gp inhibitors.

Recently, we have successfully developed a novel technique for synthesizing self‐assembled nanoparticles (NPs) using a range of natural bioactive molecules. Additionally, we have discovered that certain NPs possess the capability to serve as carriers, facilitating the delivery of other drugs to specific target tissues.^18^, ^19^, ^20^

This study aims to identify potent P‐gp inhibitors from natural bioactive molecules and develop self‐assembled NPs. These synthesized NPs possess both anti P‐gp activity and drug delivery capabilities, enabling the targeted delivery of chemotherapy drugs to the GBM and enhancing chemotherapy sensitivity. The findings of this research will provide novel insights into the utilization of efficient anticancer drugs in GBM, facilitate their clinical implementation, and revolutionize GBM treatment.

## 2 MATERIALS AND METHODS

### 2.1 Materials

Polyvinyl alcohol (PVA) was purchased from Sigma‐Aldrich LLC [USA]. Ursolic acid, betulinic acid, glycyrrhetinic acid, and oleanolic acid was purchased from Nanjing Chunqiu Biological Products Co., Ltd [purity (98%), China]. Stigmasterol and β‐sitosterol was purchased from Aladdin reagent Co., Ltd [purity (98%), China]. Coumarin‐6, rhodamine 123 and rhodamine B200 was purchased from Shanghai Yuanye Bio‐Technology Co., Ltd [China]. The antibodies included the following: anti‐P Glycoprotein [GTX108354, GeneTex, USA]; anti‐Phospho‐p44/42 MAPK (p‐ERK1/2) (Thr202/Tyr204) [#4370S, Cell Signaling Technology (CST), USA] and anti‐beta Actin [GB15001, Servicebio, China].

### 2.2 Synthesis of self‐assembled nanoparticles

In this study, we prepared terpenoids and saponins nanoparticles using the solvent evaporation method. Specifically, the natural bioactive molecule was dissolved in 1 mL of an organic solvent (ethyl acetate, N‐butanol, or dichloromethane) and added dropwise to a vigorously shaken 3 mL solution of 2.5% PVA. The resulting mixture was then sonicated and poured into a 30 mL solution of 0.3% PVA, which was continuously stirred for 6 h. Afterwards, the liquid was collected and centrifuged at 18,000 rpm for 30 min to concentrate the product. The supernatant was then discarded, and the precipitate was resuspended in 1 mL of distilled water. The suspension was centrifuged at 1000 rpm for 5 min to remove any remaining impurities, and the supernatant was retained. The supernatant was sonicated for 3 min and subsequently freeze‐dried for long‐term storage. For the synthesis of UA NPs with PTX and fluorescent dyes (coumarin‐6 and rhodamine B200), the PTX and dyes were dissolved together with UA in the organic solvent. The subsequent steps in the synthesis were the same as the separate synthesis of UA NPs.

### 2.3 Dynamic light scattering (DLS)

The NPs were diluted in double distilled water to achieve a final concentration of 0.1 mg/mL. Subsequently, the Zetasizer Nano ZSP (Malvern Instruments Ltd., UK) was used to measure the hydration diameter and zeta potential.

### 2.4 Scanning electron microscopy (SEM)

The samples were coated with gold using an auto fine coater JFC 1600 (JEOL Ltd., Japan) and then imaged using a Zeiss SIGMA field emission scanning electron microscope (Zeiss SIGMA, Carl Zeiss AG, UK).

### 2.5 Transmission electron microscopy (TEM)

The NPs (50 μg/mL) were applied onto a 100 mesh porous carbon‐coated copper grid and examined using a JEOL 1230 Transmission Electron Microscope (JEOL Co., Ltd., Japan) at 100 kV.

### 2.6 Cell Viability assay

U87MG cells were cultured in high glucose medium supplemented with 1% FBS. Afterward, the cells were treated with NPs and various concentrations of PTX for 24 h. The culture medium was then discarded, and 100 μL of fresh medium was added to each well. The cells were incubated in the medium containing 10% Cell Counting Kit‐8 (CCK‐8) for 1 h. Subsequently, the absorbance value of the samples at 450 nm was measured using an enzyme‐linked immunosorbent assay. The CCK‐8 analysis, provided by Target Mol Inc. (China), was employed to assess the anti‐proliferative activity of PTX.

### 2.7 Rhodamine 123 accumulation assay

Incubate U87MG cells with varying concentrations of NPs for 24 h. After incubation, remove the culture medium and replace it with medium containing 5 μg/mL rhodamine 123 for 1 h. Collect the cells and measure the fluorescence signal of U87MG cells using a flow cytometer from Beckman, USA. Quantitative analysis should be performed using FlowJo v10 software. The cells should then be fixed and stained with DAPI. Capture fluorescence images of DAPI and rhodamine 123 using a confocal microscope, specifically the Olympus X51 model from Japan.

### 2.8 Immunofluorescence Staining

Prepare the cell slides by fixing them with 4% paraformaldehyde for 30 min. Then, treat the slides with 0.2% tritonx‐100 for 15 min. Next, add 1% BSA to block antibodies. Apply 100 μL of primary antibody (1:100) to each slide and place them in a wet box at 4°C overnight. Incubate the second antibody (Antgene, Wuhan, China) at room temperature for 1 h. Apply DAPI anti‐fluorescence quenching agent onto a glass slide, cover it with a cover glass, and store at −20°C. Finally, image the slide using a confocal microscope (Olympus, Japan).

### 2.9 High‐performance liquid chromatography (HPLC)

The drug loading and encapsulation efficiency of UA NPs were determined using an Agilent 1100 system (Agilent Technologies, Santa Clara, USA). For ursolic acid, the mobile phase consisted of acetonitrile, ammonium acetate, and methanol in a ratio of 67:21:12. The detection wavelength was set at 210 nm, and the injection volume was 10 μL. For paclitaxel, the mobile phase comprised acetonitrile and water in a ratio of 50:50. The detection wavelength was set at 230 nm, and the injection volume was 10 μL.

### 2.10 In vitro release of UA‐PTX NPs

The in vitro release of UA‐PTX NPs was assessed using dialysis in vitro. To ensure that the drug release meets the desired leakage conditions, a PBS solution containing 1 mol/L sodium salicylate was chosen as the release medium. A pre‐swelled dialysis bag with a molecular weight cutoff (MWCO) of 3.5 kDa was used, and 1 mL of UA‐PTX NPs was added to the bag. The bag was then sealed and immersed in a flask containing 19 mL of the release medium. The release experiment was conducted in parallel using three separate parts, each shaken at 37°C and 200 rpm. At specific time points (3, 6, 12, 24, 48, 72, and 168 h), 0.2 mL of the release medium was sampled from each part. Subsequently, an equal volume of blank release medium preheated to 37°C was added to the respective parts. The concentration of PTX in each sample was determined using HPLC.

### 2.11 Fluorescent imaging

Coumarin‐6 and was using for in vivo fluorescence imaging. We perform fluorescence quantification to ensure that the fluorescence intensity between each group is consistent.UA NPs loaded with coumarin‐6 was injected intravenously through the tail vein. After 8 h, the mice were euthanized and subjected to saline irrigation. The brain and other organs were removed and imaged using the IVIS chemiluminescence imaging system. Quantify fluorescence intensity using Living Image 4.4 (Xenogen).

### 2.12 Intracranial xenograft model

The 6‐week‐old Babl/c nude mice were secured in a stereotactic apparatus and anesthetized using isoflurane. The scalp was incised and the anterior fontanel was exposed. An injection point located 2 mm in front of the anterior fontanel and 1 mm on the right side was selected. The skull was carefully drilled and the dura mater was opened. Using a micro injector, a 3 μL U87MG cell suspension (1 × 10^5^ cells/μL) was aspirated and injected at a rate of 2 min/μL. Once the injection was completed, the skull was sealed with bone wax and the scalp was sutured. The nude mice were then returned to their cage for rewarming.

### 2.13 Statistical analysis

All experiments were conducted a minimum of three times, and the data was presented as mean ± standard deviation (SD). The significance of differences between two groups was evaluated by performing a two‐tailed unpaired Student's t‐test using of SPSS software version 24. For comparisons involving multiple groups, a one‐way analysis of variance (ANOVA) was conducted, followed by a Tukey post hoc test. The normality of data was assessed using the Shapiro–Wilk test, and the data with non‐normal distribution were evaluated using the Mann–Whitney U test. All statistical charts were created using GraphPad Prism 8.0.

## 3 RESULT AND DISCUSSION

### 3.1 Screening P‐gp inhibitors through rhodamine 123 accumulation in GBM cells

**FIGURE 1:**
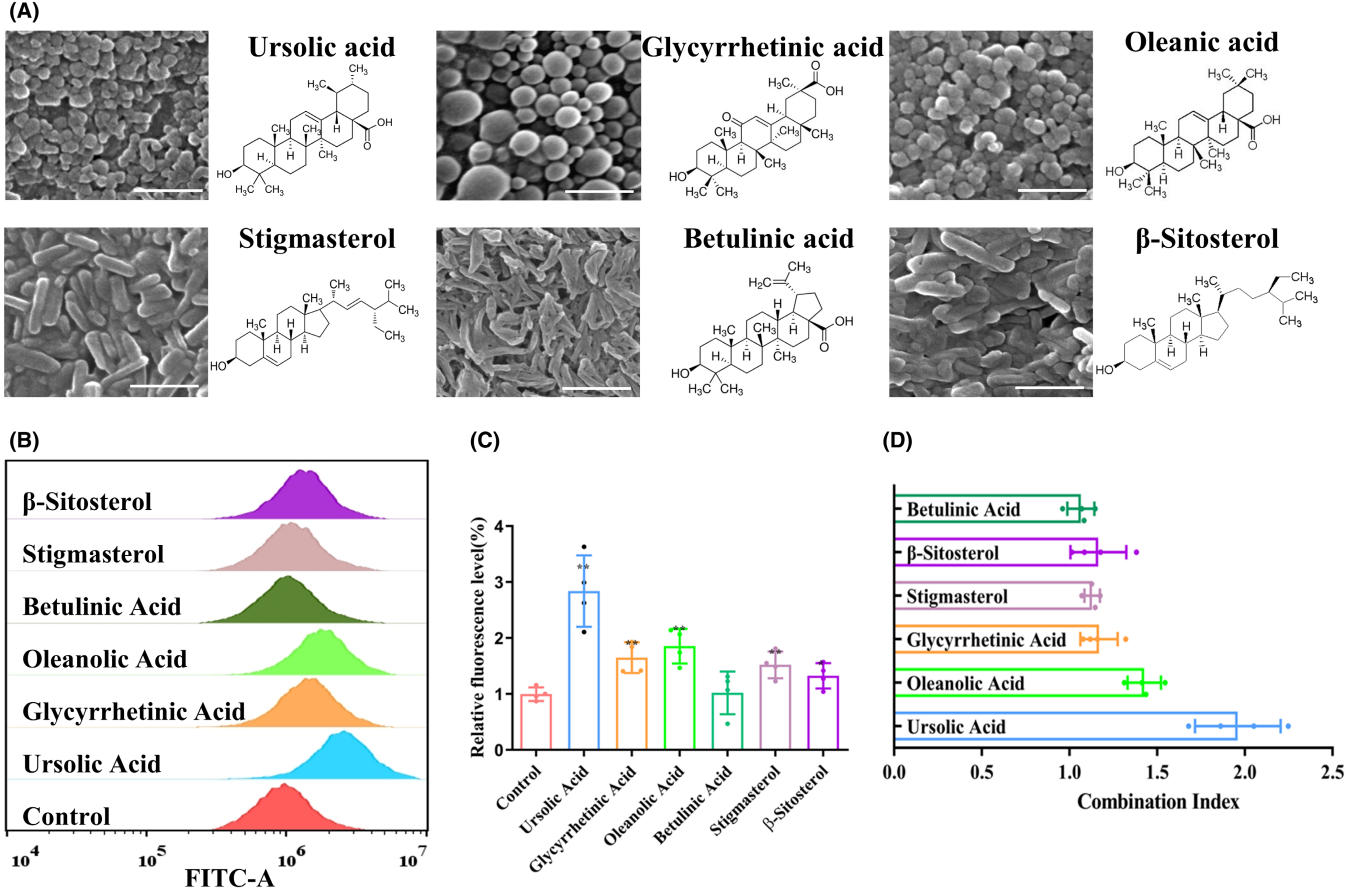
Screening P‐gp inhibitors through rhodamine 123 accumulation in GBM cells. (A) The morphology and size of 6 candidate NPs observed by SEM analysis, scale bar: 500 nm. (B, C) Rh123 accumulation in U87MG cells following treatment with 2 μM NPs by flow cytometry. (D) Combined index of natural product NPs and PTX on U87MG detected by CCK‐8 assay.

Previous studies have reported the screening of P‐gp inhibitors from a variety of natural bioactive molecules, including alkaloids, flavonoids, coumarins, resins, saponins, terpenoids, and other compounds.^21^ In this study, we focused on terpenoids and saponins as representative natural bioactive molecules and prepared NPs using the solvent evaporation method. By analyzing the size and morphology of the NPs, we identified six potential natural bioactive molecular NPs: ursolic acid NPs (UA NPs), glycyrrhetinic acid NPs (GA NPs), oleanolic acid NPs (OA NPs), stigmasterol NPs (ST NPs), betulinic acid NPs (BA NPs), and β‐sitosterol NPs (BT NPs). Scanning electron microscopy (SEM) analysis revealed that OA NPs, GA NPs, and UA NPs exhibited a relatively regular spheroid shape with diameters of 150 nm, 320 nm, and 210 nm, respectively. On the other hand, ST NPs, BA NPs, and BT NPs appeared rod‐shaped with dimensions of 450 × 110 nm, 320 × 70 nm, and 420 × 130 nm, respectively (Figure 1A).

In order to investigate the impact of P‐gp on drug efflux, it is common practice to use established cell lines for in vitro screening. This approach is crucial for subsequent in vivo research.^22^ Rhodamine 123 (Rh123) is a fluorescent dye commonly used to study the function of P‐gp. Being a substrate of P‐gp, Rh123 can be actively excreted from cells. To quantitatively evaluate the effect of P‐gp inhibitors in cells, a Rh123 accumulation assay is employed.^23^, ^24^, ^25^ In this study, we aimed to determine whether the self‐assembled NPs of bioactive molecules synthesized above affect the function of P‐gp. To assess this, we treated U87MG cells with the six candidate NPs and analyzed the accumulation level of Rh123 in the cells using flow cytometry. Our results revealed that the accumulation level of Rh123 in U87MG cells was higher in the presence of bioactive molecular NPs (2 μM) compared to the control group. Among the candidate NPs, the UA NPs group demonstrated the most significant increase in Rh123 accumulation (Figure 1B,C). In previous studies, it has been reported that certain bioactive molecules, including UA, BA, GA, ST, and BT have inhibitory effects on tumor cells.^26^, ^27^, ^28^, ^29^, ^30^ Therefore, we also investigated the inhibitory effects of these bioactive molecules on GBM and attempted to explore their combined effects with PTX. According to the results, at a concentration of 2 μM, certain bioactive molecular NPs exhibit inhibitory effects on U87MG, among which ST NPs demonstrated the strongest effect with an inhibition rate of 12.3% (Figure S1). We obtained the combined index of each groups combined with PTX, and found that the combined index of UA NPs and PTX was the highest, reaching 1.94 (Figure 1D). These findings highlight that UA NPs have the strongest synergistic effect with PTX. Based on the inhibition of R123 efflux by UA, we speculate that it may be due to UA reducing P‐gp efflux, leading to PTX accumulation and enhancing the effect of PTX. Therefore, we chose UA NPs for subsequent experiments.

### 3.2 UA NPs enhance the chemosensitivity of PTX in GBM cells

**FIGURE 2:**
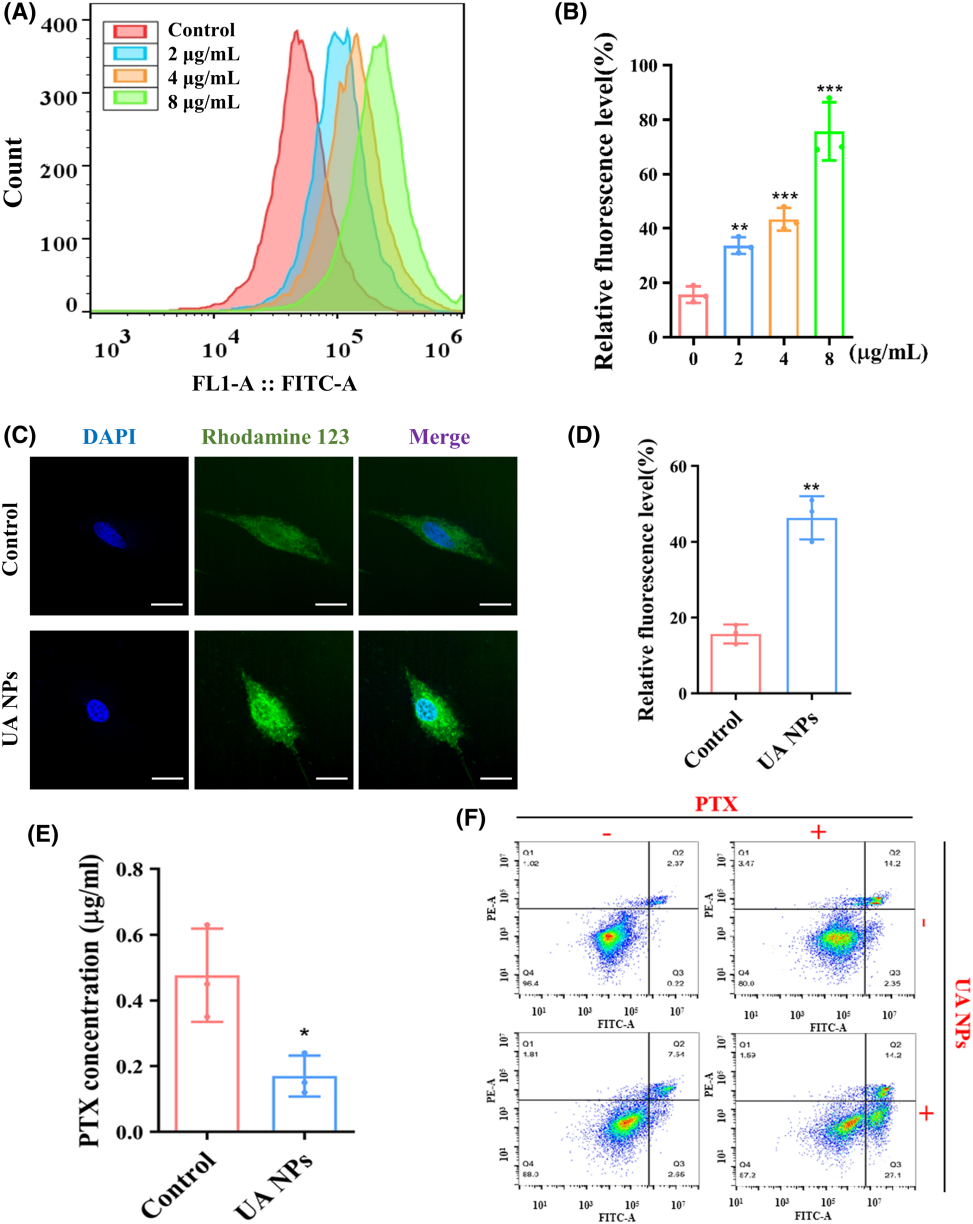
UA NPs enhance the chemosensitivity of PTX in GBM cells. (A, B) Rh123 accumulation in U87MG cells after treatment with various concentrations of UA NPs by flow cytometry. **p < 0.01; ***p < 0.001. (C, D) Rh123 uptake behavior in U87MG cells observed by immunofluorescence. scale bar: 10 μm. **p < 0.01. (E) Cell viability measured by CCK8 assay following treatment with PTX or UA NPs + PTX. (F) Apoptosis analysis of U87MG cells by flow cytometry following treatment with PTX or UA NPs + PTX.

We then treated U87MG cells with different concentrations of UA NPs and assessed the accumulation of Rh123 in the cells using flow cytometry. The results demonstrated a dose‐dependent increase in intracellular Rh123 levels (Figure 2A,B). To further clarify the inhibitory effect of UA NPs on P‐gp efflux transport function in U87MG cells, we employed confocal microscopy to observe the distribution of Rh123. The findings indicated that the incubation of UA NPs significantly enhanced the accumulation of Rh123 in the cells and inhibited the efflux transport function of P‐gp (Figure 2C,D). To further investigate the inhibitory effect of UA NPs on PTX efflux, we co‐incubated the medium containing PTX with U87MG cells. Then, the cells were co‐incubated with PBS for 1 h to detect the concentration of PTX excreted by U87MG. The results showed that in U87MG cells treated with UA NPs, the PTX efflux into PBS was significantly smaller than that in the control group. This further demonstrates the inhibitory effect of UA NPs on PTX efflux (Figure 2E). Additionally, flow cytometry analysis showed that incubation with UA NPs increased the induction of apoptosis by PTX at the same concentration in U87MG cells, further confirming the ability of UA NPs to enhance the chemical sensitivity of PTX (Figures 2F and S2). We further utilized CCK‐8 analysis to investigate the synergistic anti‐glioma effects of UA and PTX at varying UA concentrations. We observed that the synergy index consistently exceeded 1, with its peak at a UA concentration of 2 μg/mL (Figure S3). These findings suggest the chemosensitization potential of UA NPs on PTX. However, the mechanism underlying the inhibition of P‐gp function by UA NPs requires further investigation.

### 3.3 UA NPs suppress P‐gp protein expression via the ERK1/2 pathway

**FIGURE 3:**
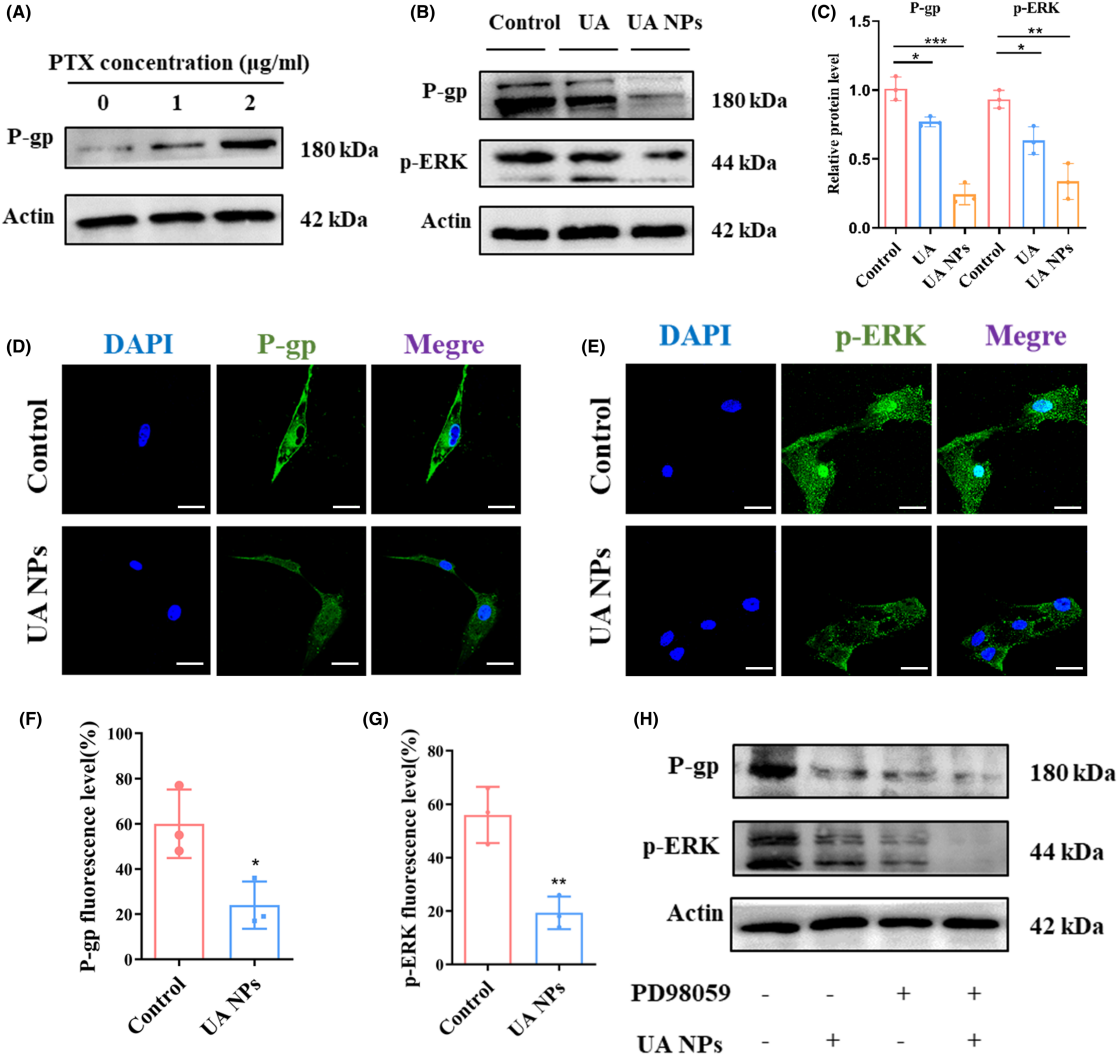
UA NPs suppress P‐gp protein expression via the ERK1/2 pathway. (A). P‐gp protein level after treatment with PTX detected by western blot. (B, C) P‐gp and p‐ERK1/2 protein level after treatment with free UA and UA NPs detected by western blot. *p < 0.05; **p < 0.01; ***p < 0.001. (D, F) P‐gp protein level after treatment with UA NPs detected by immunofluorescence. scale bar: 10 μm. *p < 0.05. (E, G) P‐ERK1/2 protein level after treatment with UA NPs detected by immunofluorescence. scale bar: 10 μm. **p < 0.01 (H) P‐gp and p‐ERK1/2 protein level after treatment with UA NPs and ERK1/2 phosphorylation inhibitor PD98059 detected by western blot.

To investigate the molecular mechanism by which UA NPs inhibit P‐gp activity, we initially examined the impact of UA NPs on P‐gp protein expression levels using western blot analysis. We observed that the incubation of PTX induced an increase in P‐gp protein expression in U87MG cells (Figures 3A and S4). However, after treatment with UA and UA NPs, there was a significant decrease in the protein expression level of P‐gp in the cells (Figure 3B,C). Additionally, we found that the phosphorylation level of ERK1/2, a core protein of the MAPK family and an important transmitter for transduction from the cell surface to the nucleus,^31^ was also significantly reduced following incubation with UA and UA NPs (Figure 3B,C). These findings were further confirmed through confocal microscopy, where we observed a reduction in the expression of P‐gp and P‐ERK1/2 proteins after treatment with UA NPs (Figure 3D–G). To establish the ERK1/2 pathway as a key regulator of P‐gp expression affected by UA NPs, we utilized PD98059, an inhibitor of ERK1/2 phosphorylation, in U87MG cells. Interestingly, we observed that the protein levels of P‐gp were no longer affected by UA NPs when ERK1/2 phosphorylation was inhibited (Figures 3H and S5), suggesting that UA NPs regulate P‐gp protein expression through the ERK1/2 pathway.

### 3.4 Synthesis and characterization of UA‐PTX NPs

**FIGURE 4:**
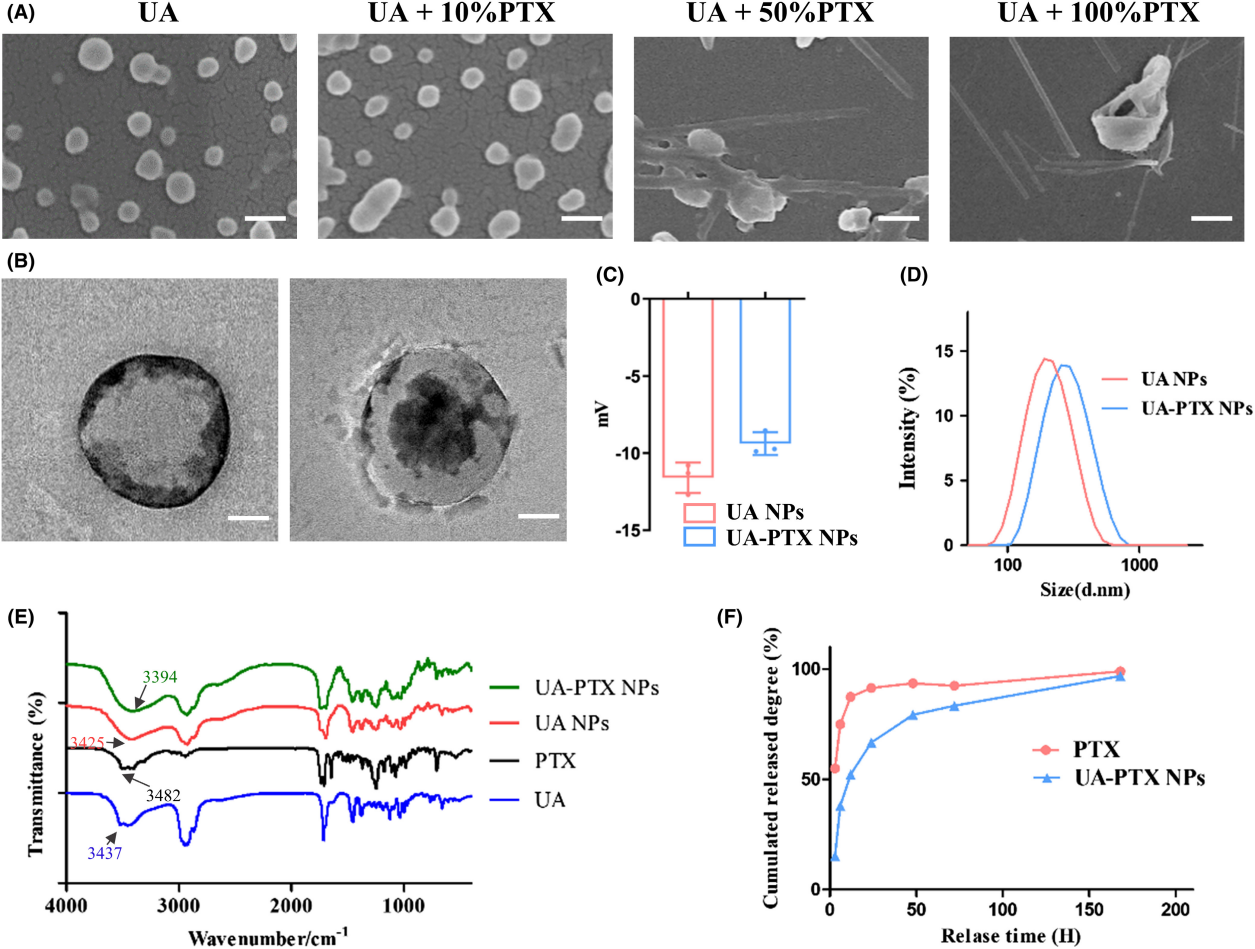
Synthesis and characterization of UA‐PTX NPs. (A) Representative SEM images of UA NPs and UA‐PTX NPs with different formulations, scale bar: 200 nm. (B) Representative TEM images of UA NPs and UA‐PTX NPs, scale bar: 50 nm. (C) Zeta potential of UA NPs and UA‐PTX NPs. (D) Hydration diameter of UA NPs and UA‐PTX NPs. (E) FTIR of UA NPs and UA‐PTX NPs. (F) Cumulative drug release profiles of UA‐PTX NPs.

In the previous study, we confirmed that UA NPs can enhance the effectiveness of PTX by inhibiting P‐gp function. To address the issues of poor water solubility and difficulty in crossing the BBB of PTX, we further encapsulated PTX in UA NPs, which have the ability to encapsulate hydrophobic small molecules for drug delivery. Through different formulations and varying ratios of PTX, we successfully obtained a series of UA‐PTX NPs products. SEM analysis revealed that when the mass ratio of PTX was less than 10%, the morphology of UA‐PTX NPs was similar to that of UA NPs, appearing spherical. However, when the mass ratio of PTX exceeded 50%, the particles grew into long rod‐shaped particles, exceeding the nanoscale, which is not suitable for intravenous administration (Figure 4A). The drug loading and encapsulation efficiency of UA‐PTX NPs were determined using high‐performance liquid chromatography (HPLC). The results showed that when the feed mass ratio of PTX was 10%, the UA‐PTX NPs achieved a maximum drug loading of 25.1% (Figures S6 and S7). Therefore, we used this ratio to prepare UA‐PTX NPs for subsequent experiments. The composition of the nanoparticles was analyzed using ^1^H NMR, and no peaks other than ursolic acid and paclitaxel were detected (Figure S8). However, due to the possibility of trace amounts of PVA not having significant peaks in ^1^H NMR, we further investigated the presence of PVA in the nanoparticles by reacting PVA with boric acid.^32^ A standard working curve (y = 0.042x + 0.016, R2 = 0.9997) was obtained by measuring the absorbance values at different concentrations of PVA. We found that PVA can be adsorbed on the surface of nanoparticles before washing, and repeated washing with distilled water significantly reduces the PVA content (Figure S9).

We used transmission electron microscopy (TEM) to observe the morphology of UA‐PTX nanoparticles (UA: PTX = 10:1) and discovered a core‐shell structure. We speculated that PTX, being hydrophobic, entered the core structure of UA NPs and remained intact during the preparation process (Figure 4B). The dynamic light scattering (DLS) analysis revealed that the average size of UA NPs was 160 nm, and the size increased to 175 nm in UA‐PTX NPs (Figure 4C). The zeta potential of UA NPs was measured to be −10.8 mV, while in UA‐PTX NPs, the potential increased to −8.78 mV due to the addition of electrically neutral PTX (Figure 4D). To further analyze the chemical structure of UA‐PTX NPs, we performed Fourier transform infrared spectrometer (FTIR) analysis. As shown in Figure 4E, the absorption peak observed at 3200‐3600 cm corresponds to the hydroxyl groups. Following the formation of UA‐PTX NPs, this hydroxyl absorption peak shifts towards the red end of the spectrum when compared to UA NPs. This shift suggests the occurrence of strong intermolecular hydrogen bonding between UA and PTX, potentially leading to self‐assembly of nanoparticles through these intermolecular interactions. As shown in Figure S10, XRD spectra of UA‐PTX NPs was recorded in the range of 8° to 80° (2 θ). In comparison to free UA, both UA NPs and UA PTX NPs spectra exhibit an absence of sharp peaks, indicating that upon the formation of nanoparticles, they transition from a crystalline phase to an amorphous state. Furthermore, differences in the XRD patterns between UA NPs and UA PTX NPs can be attributed to the incorporation of PTX within the nanoparticles. We used HPLC to study the drug release pattern of UA‐PTX NPs, as shown in Figure 4F, approximately 52% of the PTX was released at 12 h, and 83% was released at 72 h. In contrast, 88% of the free PTX was released and already in solution within 12 h. To investigate the stability of UA‐PTX NPs, we placed them in PBS for continuous stirring and periodically tested their particle size. The experimental results, shown in Figure S11, indicate that UA‐PTX NPs exhibited strong stability, with no significant changes in particle size observed during the 7‐day observation period. These results demonstrate the excellent sustained‐release effect of our nanoformulations.

### 3.5 In vitro cellular uptake of UA‐PTX NPs

**FIGURE 5:**
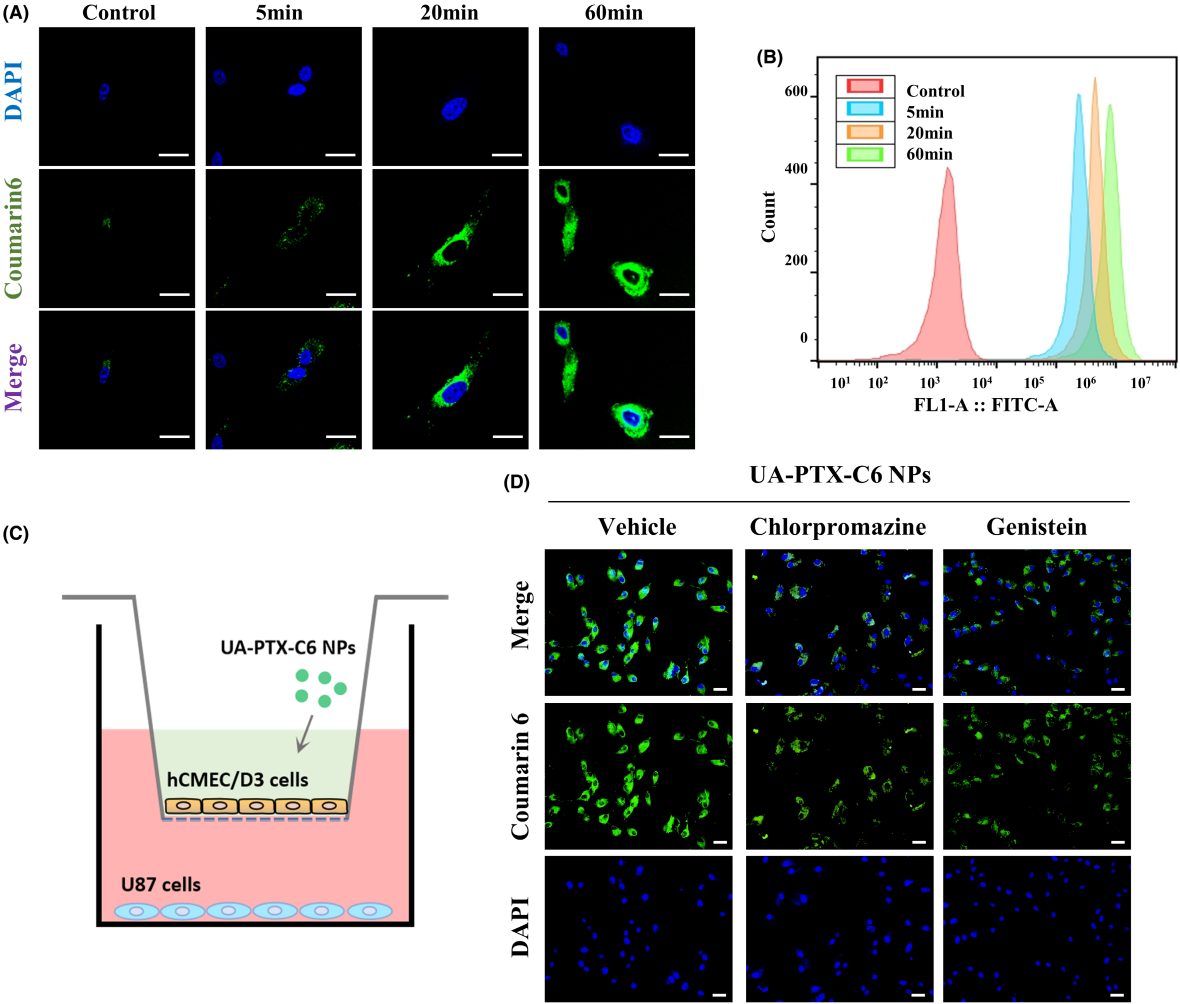
In vitro cellular uptake of UA‐PTX NPs. (A, B) Cell uptake behavior observed by fluorescence inverted microscope and flow cytometry. scale bar: 10 μm. (C) Schematic diagram of the in vitro BBB model. (D) Fluorescent image of U87MG cells after treatment with UA‐PTX‐C6 NPs and endocytosis inhibitors. scale bar: 20 μm.

In the in vitro study, we encapsulated the fluorescence dye Coumarin 6 (C6) into UA‐PTX NPs (UA‐PTX‐C6 NPs) to track the NPs within the cell. DLS detection revealed that the size and zeta potential of the NPs did not significantly change after the encapsulation of C6 (Figure S12). To investigate the uptake behavior and biological distribution of UA‐PTX‐C6 NPs in U87MG cells, we utilized confocal microscopy and flow cytometry. The results showed that after 60 min, UA‐PTX‐C6 NPs were distributed throughout the cytoplasm surrounding the nucleus, rather than within the nucleus itself (Figure 5A). These findings suggested that the internalization of UA‐PTX‐C6 NPs into tumor cells occurred in a time‐dependent manner. Flow cytometry analysis further confirmed the time‐dependent internalization of UA‐PTX‐C6 NPs into tumor cells (Figure 5B).

To understand the mechanism of UA‐PTX NPs crossing the cytoplasm, we established an external BBB model using cerebral microvascular endothelial cells HCMEC/D3. Typically, nanoparticle uptake occurs mainly through cell endocytosis, which involves clathrin and caveolin‐mediated pathways. Therefore, we incubated HCMEC/D3 cells with two endocytosis inhibitors, chlorpromazine and genistein, and examined the distribution of C6 after crossing the cells (Figure 5C). The results demonstrated that chlorpromazine inhibited the transmission of UA‐PTX‐C6 NPs in HCMEC/D3 cells, while genistein had no effect. Based on these findings, we hypothesize that clathrin‐mediated endocytosis is the mechanism by which UA‐PTX NPs cross the BBB (Figures 5D and S13).

### 3.6 In vivo biodistribution of UA‐PTX NPs

**FIGURE 6:**
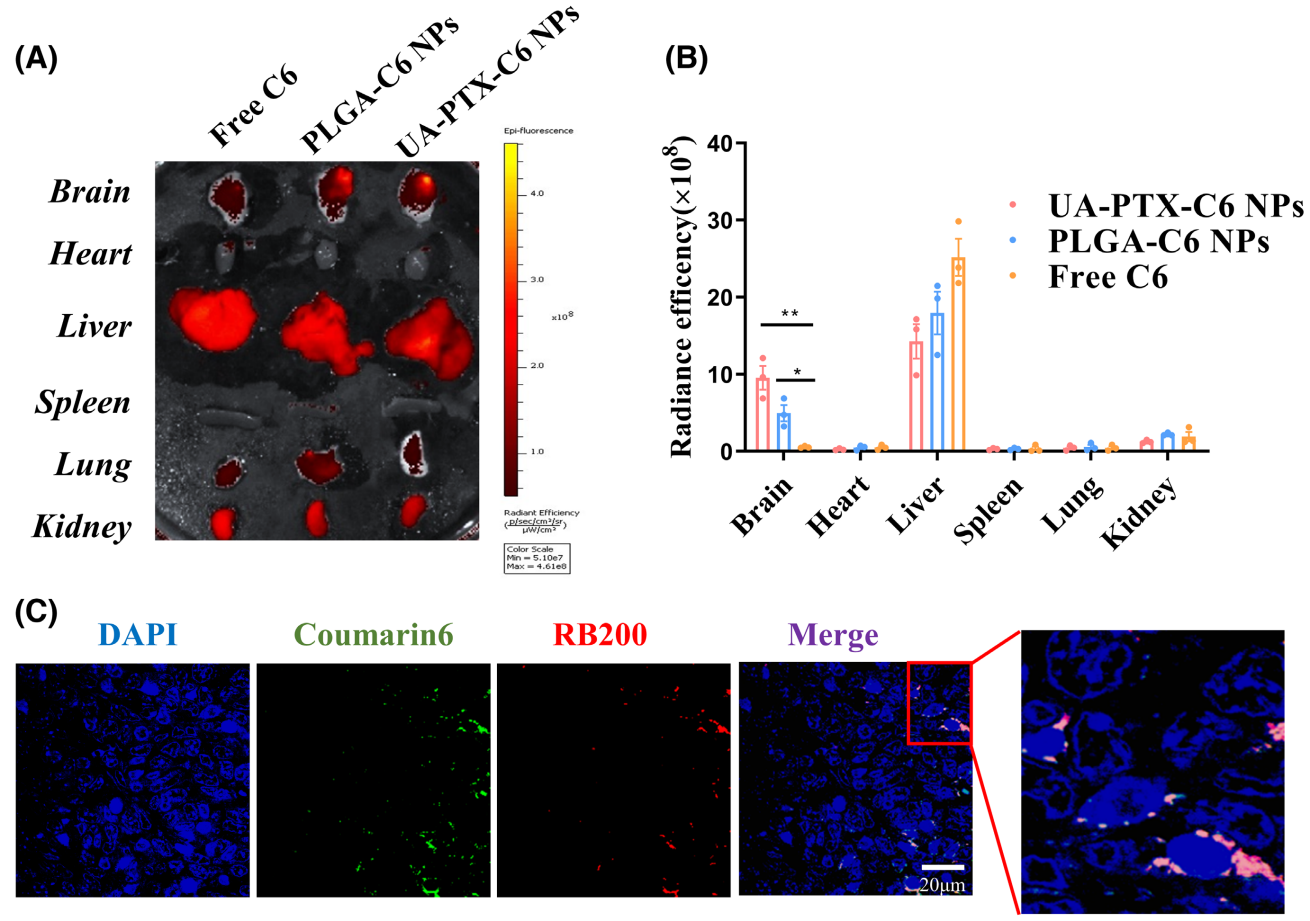
In vivo biodistribution of UA‐PTX NPs. (A, B) Ex vivo fluorescence imaging and quantification of the harvested organs by an IVIS system. *p < 0.05; **p < 0.01. (C) Representative images of C6 (green) and RB200 (red) co‐loaded UA‐PTX NPs in the regions of tumor.

We established an intracranial xenograft model to investigate the in vivo distribution of UA‐PTX NPs. After 4 weeks of tumor modeling, we injected free C6, UA‐PTX‐C6 NPs, or standard nanoparticles PLGA‐C6 NPs through the tail vein. The mice were euthanized, and the tumors and organs were collected for IVIS imaging 8 h after injection. We analyzed the fluorescence of major organs in the mice to evaluate the in vivo distribution of nanoparticles. We observed that the UA‐C6 NPs group exhibited the most intense signal in the brain tissue. By considering the location of tumor implantation, it can be inferred that the tumor tissue displayed the highest fluorescence signal. These findings suggest that UA‐C6 NP is capable of penetrating the BBB and reaching the tumor tissue. Furthermore, our results indicate that UA‐C6 NP exhibits a greater capacity for accumulating in tumor tissue compared to PLGA NP (Figure 6A,B).

To investigate the targeted enrichment of UA‐PTX NPs in GBM tissues, we prepared UA‐PTX NPs co‐loaded with two fluorescent dyes, C6 and rhodamine B200 (RB200). We encapsulated RB200 and C6, both lipid‐soluble dyes, within the same nanoparticles to prevent particle breakage, which could result in fluorescence leakage and interference from the background fluorescence of biological tissues during the experiment. Simultaneous detection of these two fluorescent components in brain and tumor tissues confirmed the presence of UA nanoparticles. After injecting the dual encapsulation UA‐PTX NPs through the tail vein for 8 h, we sacrificed the mice and obtained frozen sections of the tumor tissue for confocal microscopy. We observed that UA‐PTX nanoparticles were able to aggregate around the tumor tissue (Figure 6C). Based on the findings from the in vitro blood–brain barrier model, we hypothesize that upon injection of UA‐PTX NPs via the tail vein, they undergo clathrin‐mediated endocytosis, enabling their passage through the blood–brain barrier and uptake into the tumor tissue. The rich blood supply in the tumor tissue further enhances nanoparticle accumulation in glioma tissues. Additionally, the absence of lymphatic reflux in tumor tissue contributes to nanoparticle enrichment.

### 3.7 Enhancement of GBM xenograft model inhibition by UA‐PTX NPs

**FIGURE 7:**
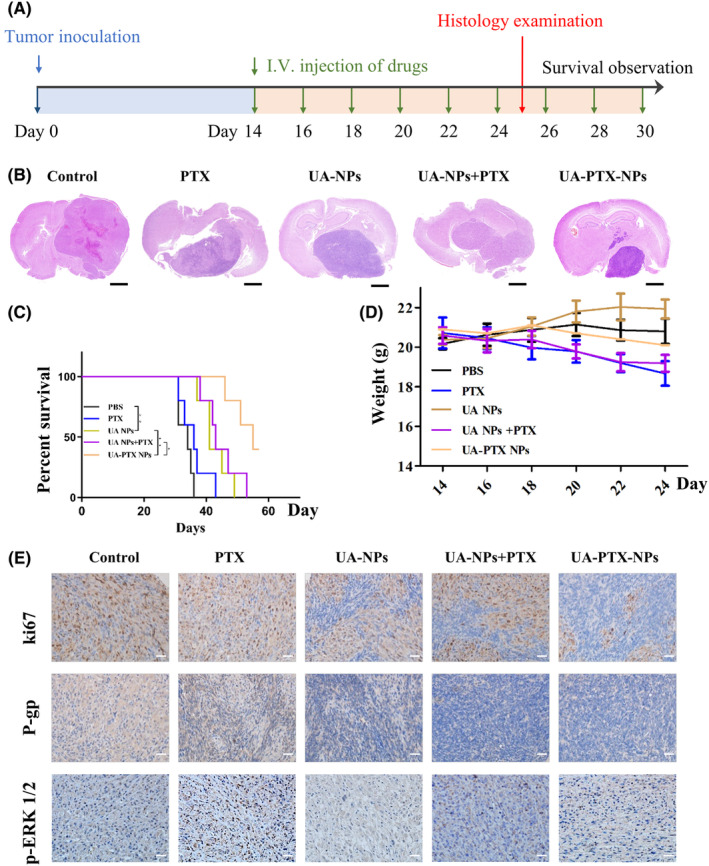
Enhancement of GBM xenograft model inhibition by UA‐PTX NPs. (A) Schematic representation of the experimental timeline. (B) HE images of the brains from nude mice bearing orthotopic U87MG glioblastoma tumors following different treatments. scale bar: 2 mm. (C) Kaplan–Meier curves showing mice survival. (n = 5). *p < 0.05; **p < 0.01. (D) Dynamic monitoring of the body weight of mice. (E) IHC staining images for Ki67, p‐ERK1/2 and P‐gp in the dissected tumors. scale bar: 50 μm.

We subsequently established an intracranial xenograft GBM model to investigate the in vivo anti‐GBM effect of UA‐PTX NPs. The experimental procedure is illustrated in Figure 7A. The drugs in each group were administered via tail vein injection three times a week. Two weeks after the initial injection, two mice from each group were sacrificed, and the size of intracranial tumors was assessed using hematoxylin–eosin (HE) staining. Our findings revealed that free PTX had only a marginal therapeutic effect on tumor growth, likely due to its inability to penetrate the BBB. Conversely, the tumor size in the UA NPs group was significantly smaller than that in the PTX group, suggesting that UA NPs possess inherent anti‐GBM properties. Lastly, compared to the UA NPs + PTX group, the tumor size in the UA‐PTX NPs group was smaller, indicating that PTX encapsulated within UA NPs can exert a more potent anti‐GBM effect (Figure 7B). The remaining mice were monitored for survival time. Consistent with the GBM size, the survival curve also demonstrated a significant therapeutic effect of UA‐PTX NPs compared to UA NPs + PTX (Figure 7C). Overall, UA‐PTX NPs have demonstrated effective treatment potential for GBM.

We conducted further investigation into the toxicity and biocompatibility of UA‐PTX NPs. The body weight of mice was continuously monitored throughout the drug treatment period. The results revealed that mice treated with PTX and UA NPs + PTX experienced a higher degree of weight loss compared to the other groups. However, the weight loss observed in the other treatment groups was not significant (Figure 7D). These findings suggest that encapsulating PTX into UA NPs can effectively reduce the toxicity associated with PTX itself. To assess the impact of UA‐PTX NPs on mouse organs, we performed HE staining. The results indicated that after treatment with UA‐PTX NPs, no pathological lesions were observed in the heart, liver, and kidneys of the mice (Figure S14). This demonstrates the excellent biocompatibility of UA‐PTX NPs in vivo.

Finally, immunohistochemical analysis was conducted on the tumors removed from the mice treated with different formulations. Consistent with the western blot results, the immunohistochemistry analysis confirmed that the treatment with UA NPs led to a significant reduction in the levels of p‐ERK1/2 and P‐gp in tumor tissue in the UA NPs, UA NPs + PTX, and UA PTX NPs groups. Additionally, the UA‐PTX NPs group showed a significantly lower ki67 compared to the UA NPs and UA NPs + PTX groups, further supporting the positive therapeutic effect of UA PTX NPs (Figure 7E).

## 4 CONCLUSION

In this study, we conducted a screening of bioactive molecular nanoparticles and identified UA NPs as effective inhibitors of P‐gp transporters. Based on this discovery, we prepared co‐assembled UA NPs embedded with PTX, which not only possessed anti P‐gp activity but also served as drug delivery vehicles. Subsequently, we investigated the properties of these NPs both in vitro and in vivo. The UA‐PTX NPs demonstrated improved water solubility of PTX, facilitated its passage across the BBB, and enhanced the sensitivity of PTX by inhibiting P‐gp function. These findings highlight the promising potential of UA‐PTX NPs in the treatment of GBM. These findings highlight the promising potential of UA‐PTX NPs in the treatment of glioblastoma (GBM). Moreover, this study introduces novel research directions for the application of anticancer drugs in GBM and represents a significant breakthrough in GBM treatment.

## AUTHOR CONTRIBUTIONS

Gang Deng, Jingbing Zhou and Qianxue Chen designed this study. Yong Li, Qingyu Zhao, Xinyi Zhu, Long Zhou, Ping Song, Baohui Liu and Daofeng Tian performed the data and wrote the paper. All authors read and approved the final manuscript.

## FUNDING INFORMATION

This work was supported by the National Natural Science Foundation of China (No. 82001311) and the Knowledge Innovation Program of Wuhan‐Shuguang Project (No. 2022020801020483).

## CONFLICT OF INTEREST STATEMENT

All authors declare that they have no competing interests.

## Supporting information


Data S1.

## Data Availability

The data used to support the findings of this study are available from the corresponding author upon request.
