# Supplementary material for: Self‐Assembled nanoparticles of natural bioactive molecules enhance the delivery and efficacy of paclitaxel in glioblastoma
Source: CNS Neurosci Ther. 2023 Dec 4;30(4):e14528. doi: 10.1111/cns.14528 (PMC11017454; doi:10.1111/cns.14528)
Supplement: Supplementary file 1 — Data S1. [file CNS-30-e14528-s001.pdf]

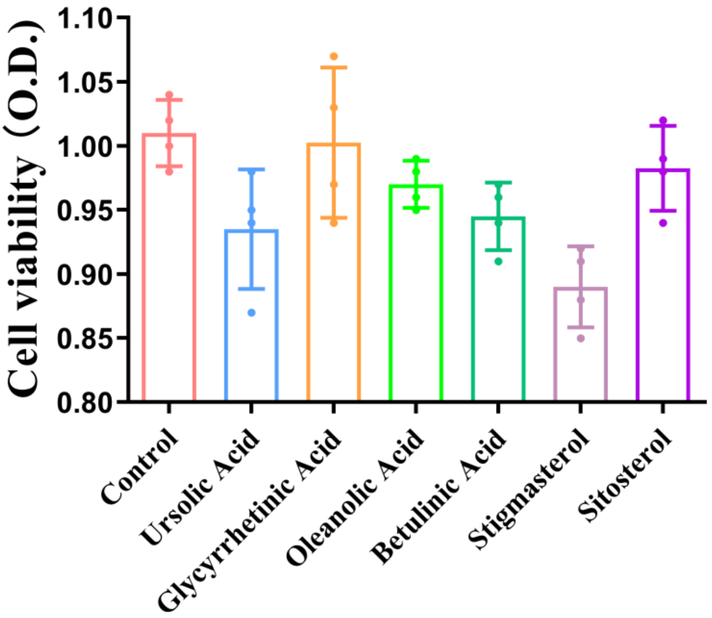

Figure S1. Cell viability of U87MG cells in 2μM certain bioactive molecular NPs.

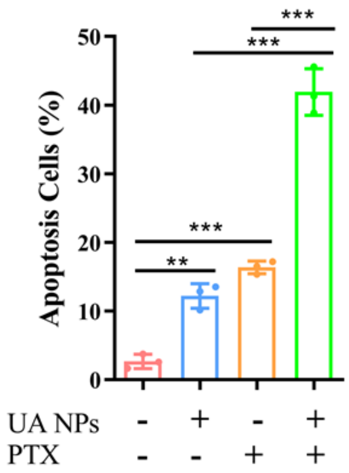

Figure S2. Apoptosis analysis of U87MG cells by flow cytometry after PTX or UA NPs+PTX treatment. **P < 0.01, ***P < 0.001.

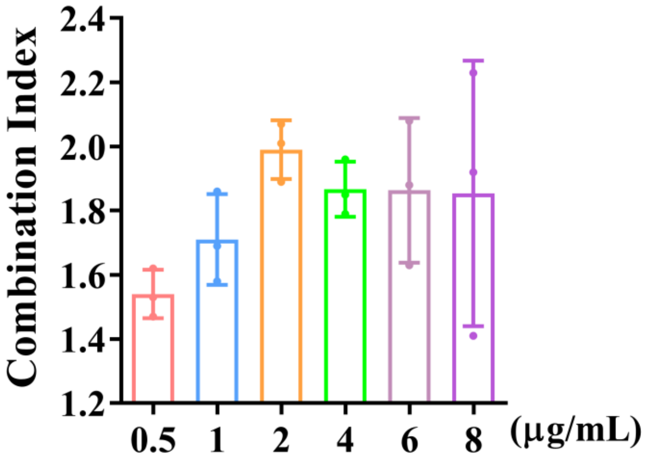

Figure S3. Combined Index of different concentration of UA.

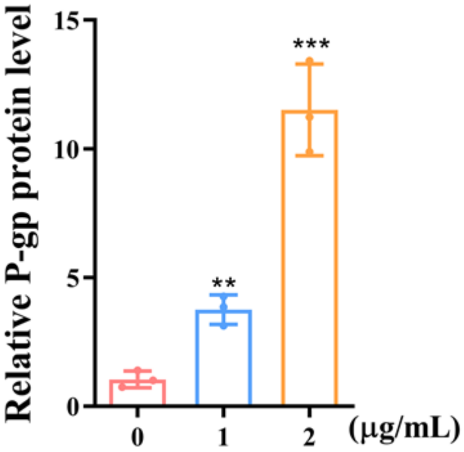

Figure S4. P-gp protein level after treatment of PTX. **P < 0.01, ***P < 0.001.

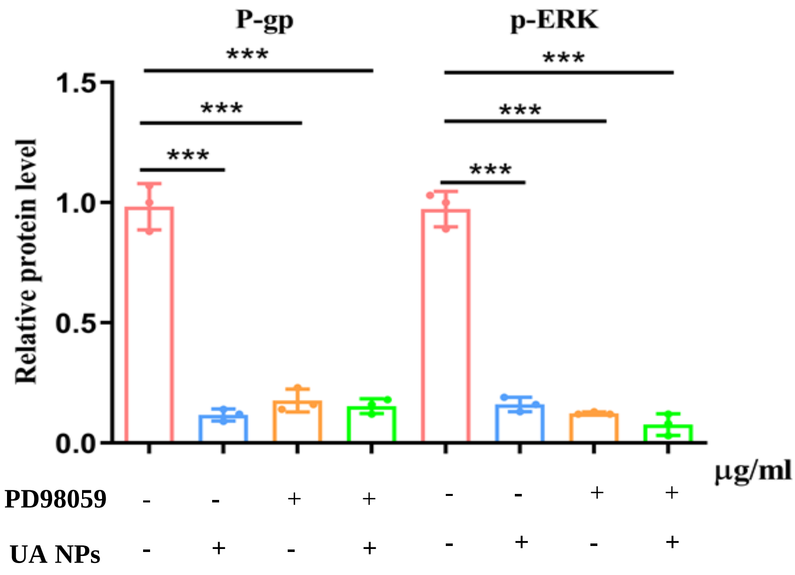

Figure S5. P-gp and p-ERK protein level after treatment of UA NPs.

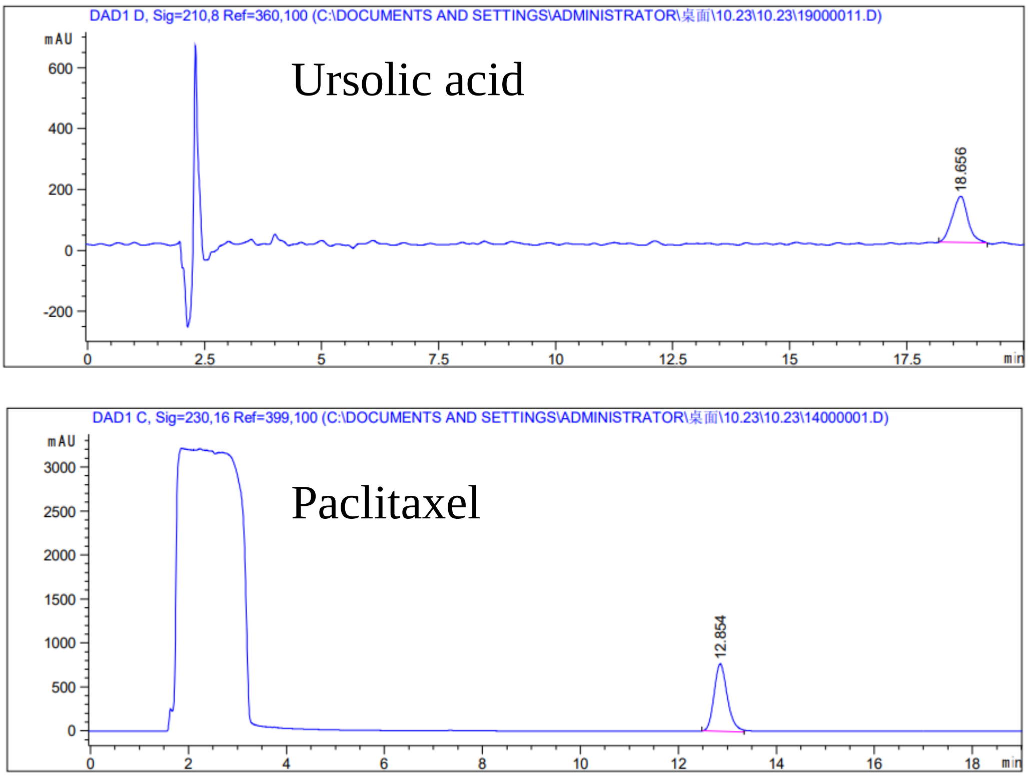

Figure S6. Identification of ursolic acid and paclitaxel by HPLC.

Formula	1	2	3	4	5
**UA (mg)**	10	10	10	10	10
**PTX (mg)**	0	0.2	0.5	1	5
**Productivity (%)**	40.7	35.0	39.2	36.1	24.3
**Drug loading (%)**	/	5.6	17.1	25.1	125.2
**Entrapment efficiency (%)**	/	39.5	53.7	36.4	24.4

Figure S7. Drug loading and entrapment efficiency of UA-PTX NPs.

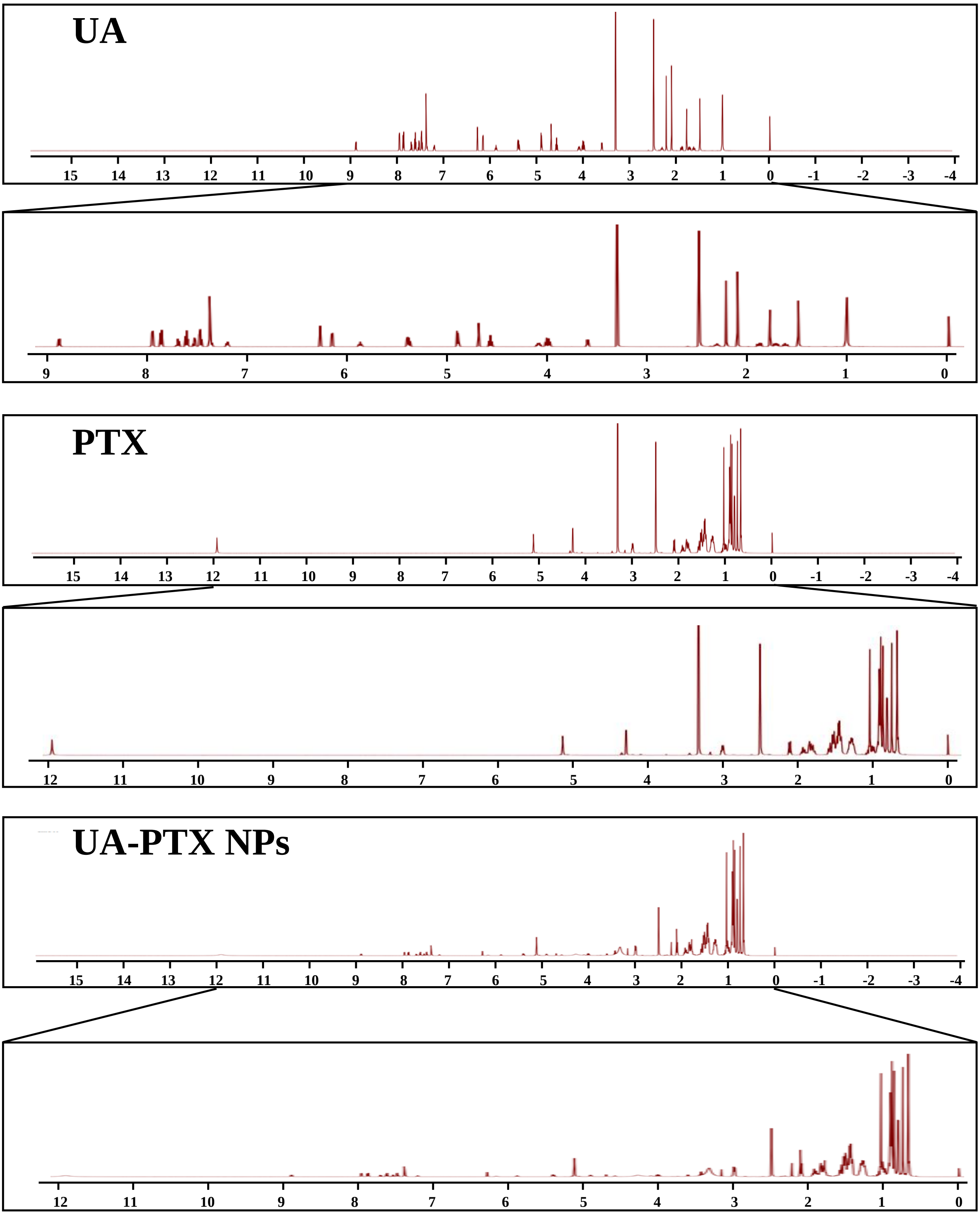

Figure S8. ^{1}H NMR hydrogen spectrum of UA and UA NPs.

A

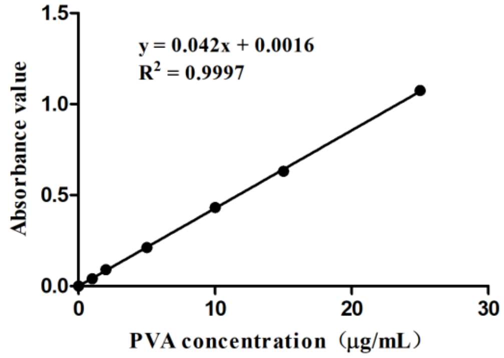

B

Group	PVA concentration (μg/ml)									
**Blank Control**	0.16	0.24	-0.05	-0.17	0.19	0.24	0.01	0.11	-0.13	-0.03
**UA-PTX NPs no washed**	1.25	1.49	2.13	0.51	0.47					
**UA-PTX NPs washed with distilled water**	0.33	0.13	0.19	-0.23	0.17					

Figure S9. Estimation of polyvinyl alcohol. A. Standard curve for PVA content measurement. B. PVA concentration of Blank Control, UA NPs no washed and UA NPs washed with distilled water.

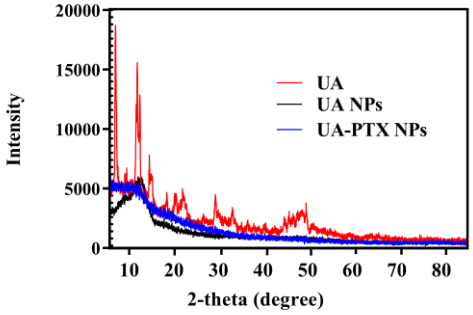

Figure S10. XRD of UA and UA NPs.

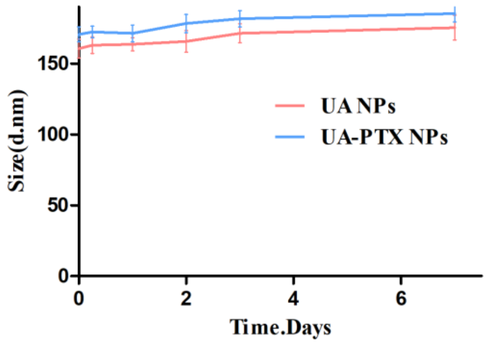

Figure S11. The stability of UA NPs in PBS.

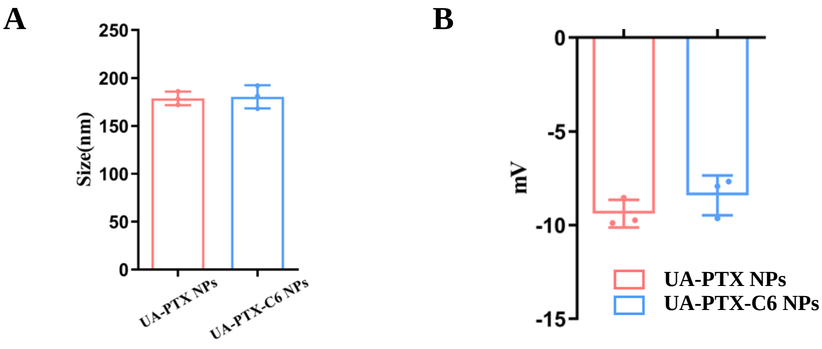

Figure S12. The size (A) and zeta potential (B) of UA-PTX-C6 NPs.

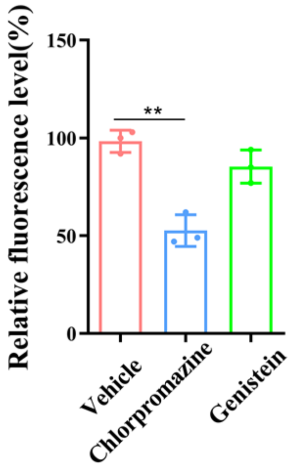

Figure S13. Quantification of U87MG cells after UA-C6 NPs processing. **P < 0.01.

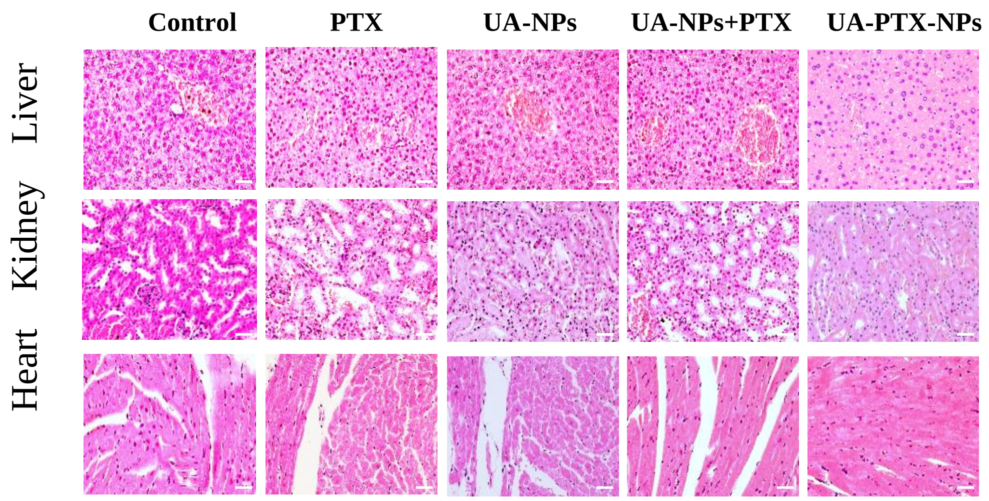

Figure S14. HE staining of heart, liver, and kidney from mice. scale bar: 50 μm.
